# Visualization of complicated fractures by 3D-printed models for teaching and surgery: hands-on transitional fractures of the ankle

**DOI:** 10.1007/s00068-022-01879-1

**Published:** 2022-02-05

**Authors:** Jonas Neijhoft, Dirk Henrich, Katharina Mörs, Ingo Marzi, Maren Janko

**Affiliations:** Department of Trauma, Hand and Reconstructive Surgery, Goethe University Frankfurt, Theodor-Stern-Kai 7, 60590 Frankfurt, Germany

**Keywords:** Teaching, Training, Fracture models, Transitional fractures 3D printing, Traumatology

## Abstract

**Aims:**

Understanding the orientation of fracture lines and mechanisms is the essential key to sufficient surgical therapy, but there is still a lack of visualization and teaching methods in traumatology and fracture theory. 3D-printed models offer easy approach to those fractures. This paper explains the use of the teaching possibility with 3-dimensional models of transitional fractures of the ankle.

**Methods and results:**

For generating 3D printable models, already obtained CT data were used and segmented into its different tissues, especially parts concerning the fracture. After the segmentation process, the models were produced with FFF (fused filament fabrication) printing technology. The fracture models then were used for hands-on teaching courses in AO course (Arbeitsgemeinschaft für Osteosynthesefragen) of pediatric traumatology in 2020 in Frankfurt. In the course fracture anatomy with typical fracture lines, approaches, and screw placement could be shown, discussed and practiced.

**Conclusion:**

The study shows the use of 3D-printed teaching models and helps to understand complicated fractures, in this case, transitional fractures of the ankle. The teaching method can be adapted to numerous other use cases.

**Supplementary Information:**

The online version contains supplementary material available at 10.1007/s00068-022-01879-1.

## Introduction

Understanding of the orientation of fracture lines and mechanism is the essential key to sufficient surgical therapy. Various classification systems, like AO (Arbeitsgemeinschaft für Osteosynthesefragen) [1] or Aitken [2], respective Salter-Harris classification [3] exist not only to classify but also to give advice on therapy options. Fractures must be correctly assigned to the right subgroups, but in the case of complicated fractures like transitional fractures of adolescent children, this can be challenging.

Usually, students and young interns are learning the characteristics of fractures and their mechanisms from books or drawings, however, the 3-dimensional (3D) understanding of these fractures is difficult with educational material that is limited to two dimensions. This is in part due to the irregular lines of different fractures [4–6]. Whereas in anatomical learning hands-on courses are common [7] and furthermore 3-dimensional visualizations are coming up [8, 9], there still is a lack of these learning methods in traumatology and fracture theory.

Using 3D-printed models in the learning process will fill this gap, which could help students not only to see but also to feel the fracture. The fused filament fabrication (FFF) 3D printing technology offers a promising possibility to cost-efficiently build and reproduce fracture models for teaching purposes from daily CT (computer tomography) data [10].

For hands-on experience, a model of transitional fractures of the ankle was chosen. These fractures, which are characteristic for young adults, offer very homogenous but complicated fracture lines. The different fractures can be divided into two- and triplane I and II:

Transitional fractures only occur in the youth or adolescence due to the open growth plate, which starts to fuse around an age of 12 years. This affects fracture lines a lot [11]. A first fraction is the twoplane fracture: The fracture is following the growth plate towards the lateral side of the ankle but stays in the same plane. While in a triplane fracture—as the name suggests—an additional plane gets involved, which is led by a metaphyseal fragment. Inside these a distinction can be made between two types: In case of triplane I, the fragment ends within the growth plate while in case of triplane II the fracture line follows through posterior epiphysis [11]. This exemplifies how important the exact understanding of this fracture is, because it is needed for the correct assignment of the CT data to the fracture classification subgroup and choice of the right procedure or conservative therapy [11–14]. Alternatively, an MRI (magnetic resonance imaging) of the distal tibia might be useful as well. Importantly, the transverse view of the fracture can only be seen in 3D imaging in contrast to plane x-rays in two views [11, 13–17].

This paper aims to show a first hands-on transitional fracture 3-dimensional models to demonstrate the options of this new technology for teaching and surgical planning.

## Methods

For building a 3D printable model, a 3-dimensional dataset of fractures as CT scans, in case of children low dose CT scans or MRI-datasets, are necessary.

The 3D printer needs a coordinate-based filesystem, so the Digital Imaging and Communications in Medicine (DICOM) data must be segmented into its parts of interest and then translated into a coordinate-based language, readable by the 3D printer. For segmentation, the software called 3DSlicer (*The Slicer Community*, Version 4.11) was used, but there are various software solutions available. The finer and narrower the slices of the CT scans are (e.g. Array CTs) the more detailed the resulting model gets and the less interpolation has to be done.

**Fig. 1:**
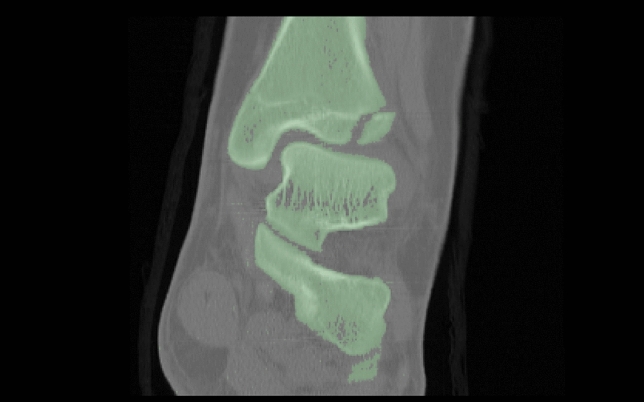
Example of a threshold-based segmentation. The voxels are assigned to one group (green) using different HU-borders

**Fig. 2:**
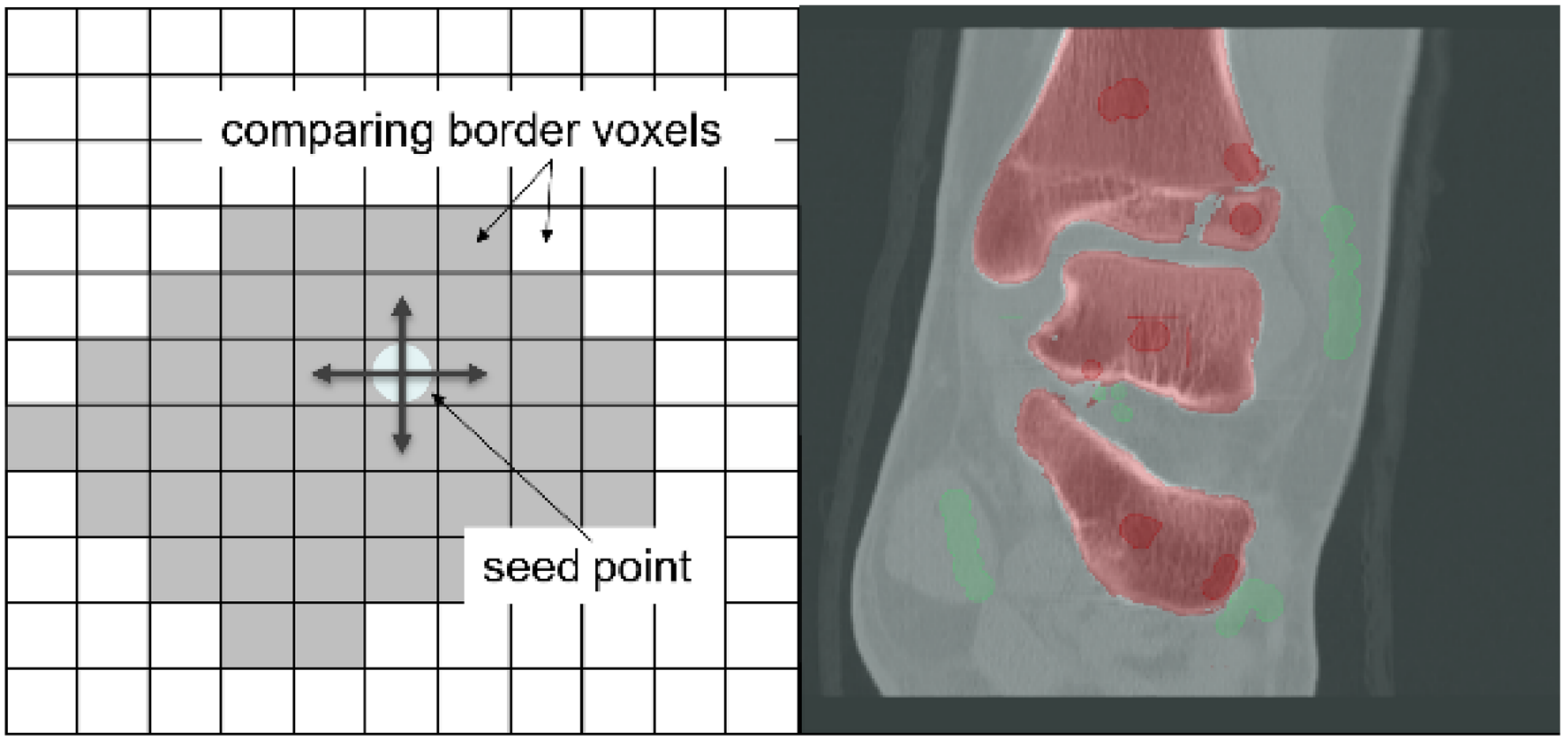
Left: A schematic overview of region growing: Seed points are placed within the desired regions. The voxels surrounded by the seed point are compared and are included or excluded to the different groups. Right: The placed dark red seed points are assigned to the red group of interest, while the chosen voxels in green are the basis for the light green group

First the bone of interest must be separated from soft tissue. Therefore, a combination of two methods was used: The threshold-based method and region growing. The threshold-based method uses different defined boundaries deriving from the Hounsfield units (HU) of bone (around 500–1500 HU), soft tissue and blood. It assigns every single voxel depending on its HU parameter to one of these groups (Fig. 1). For region growing one must define landmarks and regions of interest as well as the surrounding—for this case—irrelevant tissue. Inside these marked areas every border voxel is compared with its surrounding voxels. Depending on the defined limitation the algorithm decides whether to include or exclude this voxel. In case it was included in further steps it again gets compared to the next one. Thereby the region is growing (Fig. 2). Through further limitation of these boundaries and manual addition or exclusion of wrong assigned voxels the step of segmentation is finished.

Then segmented bone can be exported as a standard tessellation language (STL) file, which is broadly used for a lot of printers and slicing software. The file then is processed by another software for further adjustments in terms of printability like adding manual support structures, such as *Meshmixer* (*Autodesk*, Version 3.5.474). After that, the file is machined by a slicer. This software works as the last step before printing and is translating the STL file into a coordinate-based file format, which is called GCODE. In the present case, Cura (*Ultimaker,* Version 4.8) was used. The printing parameters, which are depending on different aspects like level of details or printing time, are defined here too. A very basic overview of the parameters used for the translational fractures from this study are shown in Table 1. For printing a slightly modified CR-10 Pro (*Creality,* Shenzhen, China) was used.

**Table 1:** Overview of some printing parameters

Parameter	Value
Layer height	0.24 mm (for faster printing)
Infill density	10% (or above)
Top Layers	3
Wall line count	3
Support overhang angle	80°

These always need to be adjusted to the different printer and model itself

Thereafter the model can be printed as many times as needed and in different materials.

In this study, the typical two- and triplane fractures of adolescents were printed in polylactic acid (PLA) and used first in a hands-on course at the AO course of pediatric traumatology in 2020 in Frankfurt to show different subgroups and to explain therapy. Besides the understanding of the fracture types, the typical reduction and stabilization with cannulated screws was performed (Fig. 6), so that the participants will completely understand reduction and stabilization as well.

## Results

**Fig. 3:**
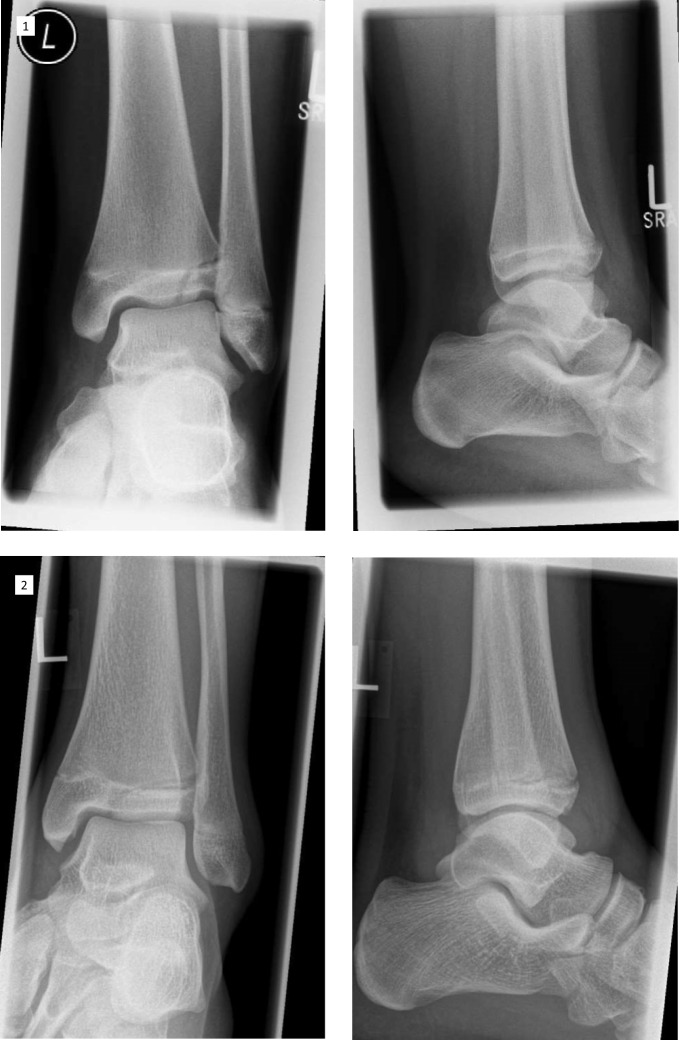
Preoperative X-Ray of Twoplane (1) and Triplane II (2). Due to medical referral, there exists no preoperative x-ray of Triplane I shown in this study

**Fig. 4:**
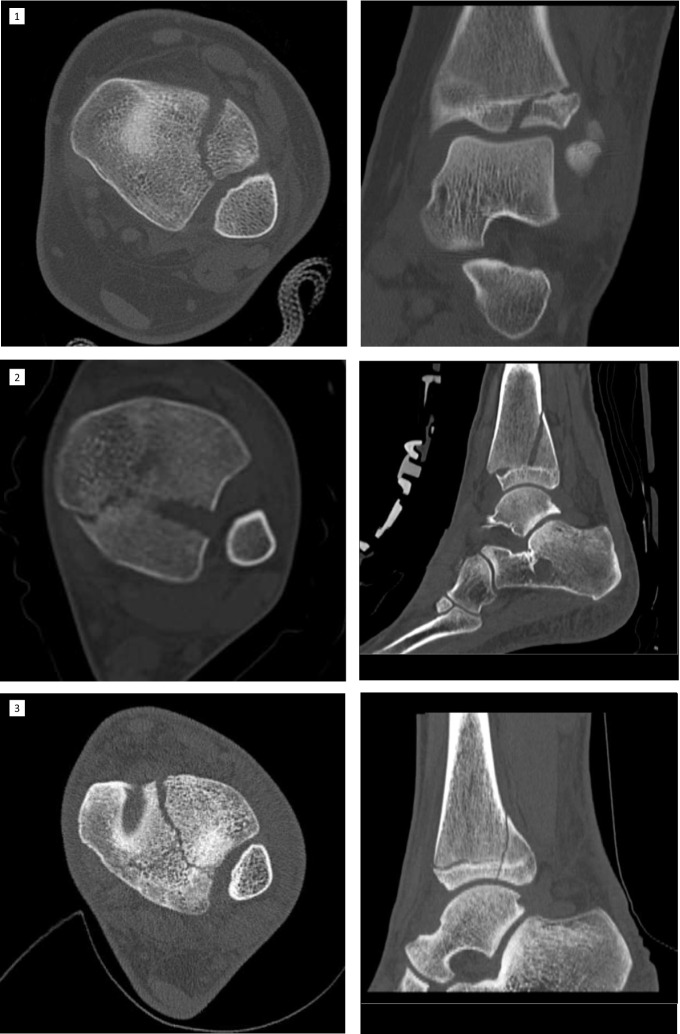
Preoperative CT images. The fracture line can be distinguished exactly by CT or MRI. Here are shown CT images of Twoplane (1), Triplane I (2) and Triplane II (3) fractures

**Fig. 5:**
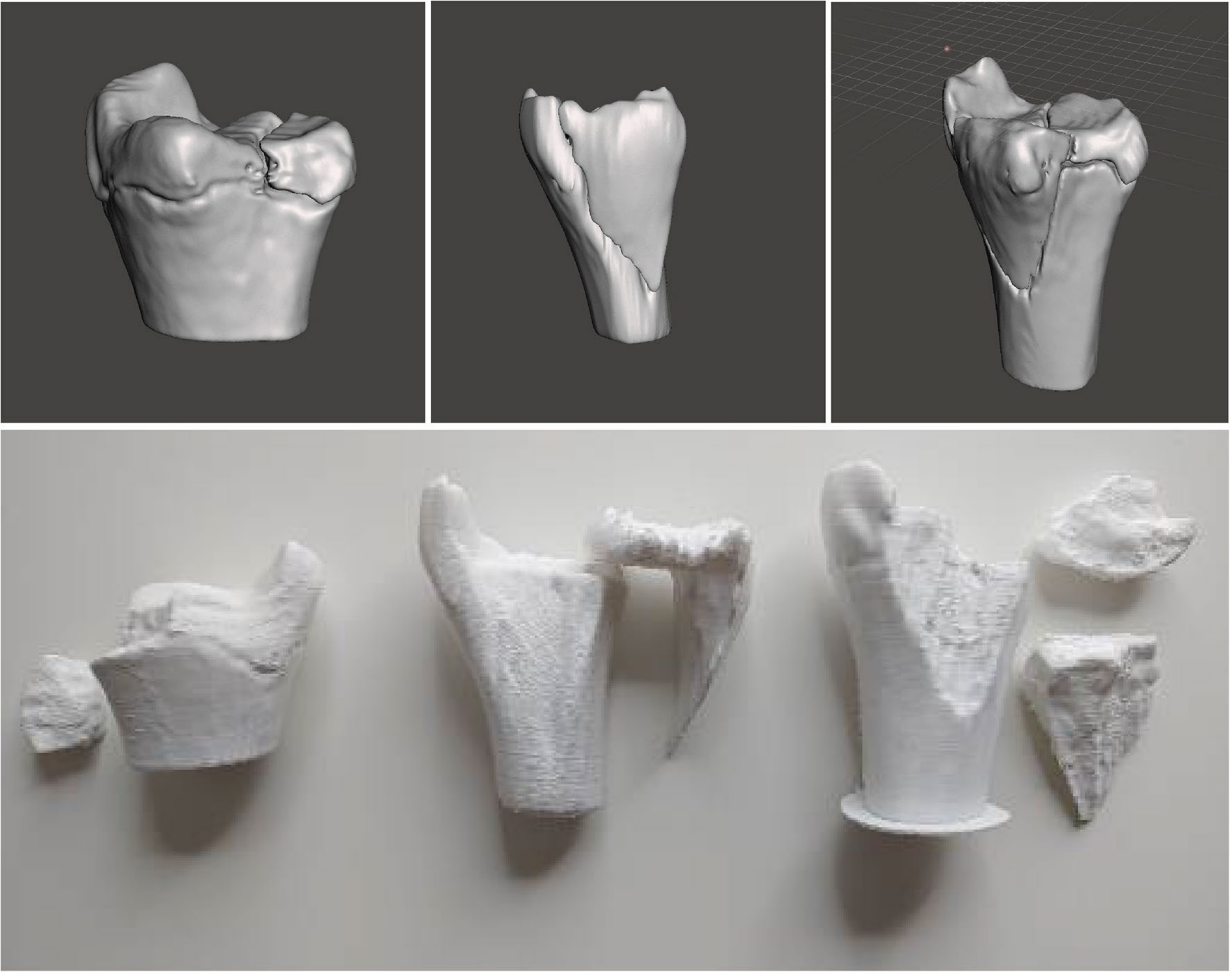
The resulting three different models in an animated figure and below with its printed life-sized model. Left: Twoplane, Middle: Triplane I, Right: Triplane II Fracture

**Fig. 6:**
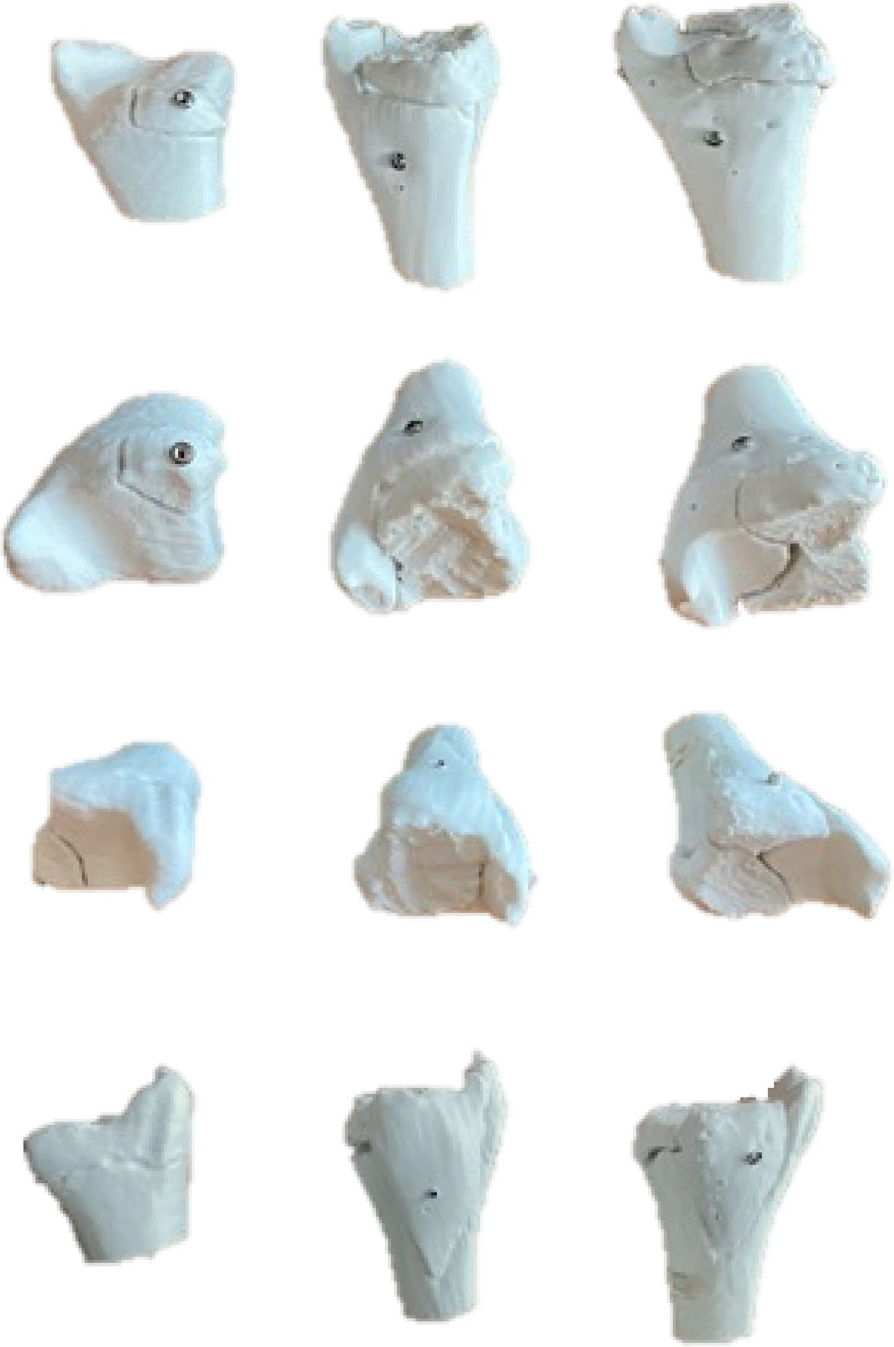
During the AO course of pediatric traumatology, correct placement of the screws was practiced. On the left is shown Twoplane, on the middle Triplane I and on the right Triplane II fracture

As already mentioned, the fracture line in the case of the twoplane fracture follows the growth plate to the lateral side in one plane. The fragment often can be seen in the x-ray on the a.p. image as seen in Figs. 3 and 4 in the CT scan. The life-sized printed models as well as the digital reconstruction is shown in Fig. 5. In most cases closed reposition is possible followed by fixation with an Kirschner´s wire or screw as seen in Fig. 6.

**Fig. 7:**
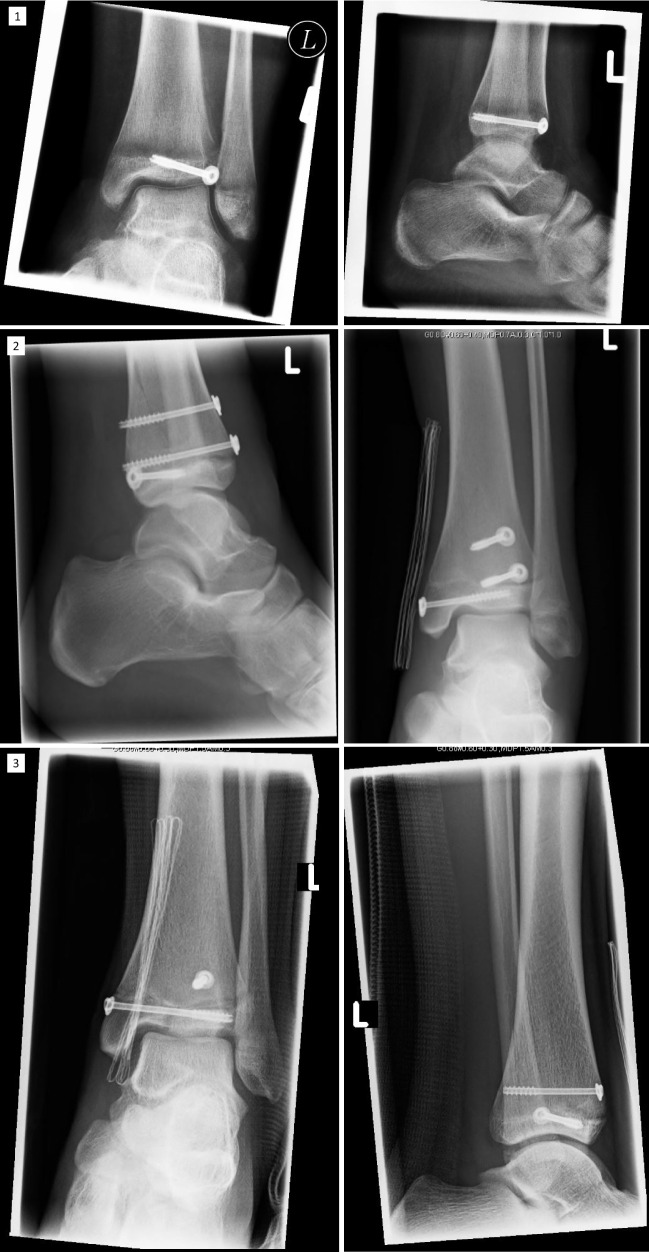
The postoperative control of screw placement of the Twoplane (1), Triplane I (2) and Triplane II (3) fracture

Pointing out fracture lines and differentiation between triplane I and II can be hard in the x-ray image (Fig. 3), as the fragment especially in lateral view can be overlayed by the fibular bone. In these cases and for planning of the surgical procedure CT- or MRI-scans can be crucial. After repositioning of the fragments in opened or closed way, screw placement is essential: The lateroventral fragment needs to be fixated with a screw so there is one resulting bone bloc, which is screwed to the tibia afterward (Fig. 6). Postoperative x-ray images of all above procedures can be seen in Fig. 7.

As different surgical approaches and procedures are necessary for each type of transitional fracture, the understanding of the fracture’s mechanism is essential. By getting hands-on with the 3D-printed models and examining them, comprehension can be improved. After theoretical understanding of the fracture, all these described procedures can be easily practiced with the same 3D-printed models provided. This hands-on can also emphasize how important correct placement of the fragment and screws is to prevent articular displacement with resulting arthrosis.

## Discussion

Printing life-sized fracture models gives the advantage of holding and feeling the model with one´s hand, instead of just looking at it. Especially in case of complicated fractures, comprehension of the fracture line is essential to the patient´s outcome. A 3d printed model can help to understand, classify and plan surgical procedure. So there are multiple advantages, not only in education but, as shown in other studies with different fracture types, also in the potential reduction of time of the surgical procedure or intraoperative blood loss [18].

Taking the fracture fragments apart allows having a look from every side and understand therapy options, surgical approaches and screw placement. Afterward, the fracture can be puzzled together again.

We used transitional fractures as an example to show the potential use of modern teaching techniques. It is even possible to try screw placement or cutting with these models, but in this case cooling of the drill/saw is essential as the plastic can melt when reaching higher temperatures on the tip of the drill. One downside is that PLA as the material used for this study, does not entirely feel like bone or artificial bone in the case of drilling, which not only is caused by the material but also the missing microstructure of cancellous and cortical bone. Perception and haptic sensation of real bone, also during drilling can be added in further studies by adding microstructure inside the models.

There exists a great variety of software for segmentation of medical data and there are numerous ways to do the segmentation, but there are some downsides that make the preparation of a model harder: Especially in case of loss of bone mass or artifacts in the CT data, region growing as well as the threshold-based method can cause wrong definitions and include broader or steeper areas with surrounding artifacts. Manual adjustments or changes in boundaries and limitations must be done, to correct the region growing algorithm. The manual correction is time-consuming, but as the algorithms are improved the results are getting better.

Although the STL file format is limited in terms of editability it offers some advantages: On one hand the file system can be read and used by lots of slicing software. This leads to the advantage of broad usability not only in FFF-printers but also SLA (Stereolithography) and other printing technologies. Furthermore, STL files offer small file sizes, which makes the distribution via the internet much easier. The 3D-printed models must not be shipped but can be printed in-house e.g. in universities or nursing schools directly for the courses, in which the used plastics in most types can be disinfected with standard surface disinfectants [19]. Despite there being some basic parameters provided for printing, these strongly depend on the printing technology, different printer brands, the material and even the models, which means these parameters must be customized individually.

Especially because the models were intended for educational use, we used CT data from our database and no CT scan was done just for the reason of printing any models. For the first models the STL datasets will be available for download. It is planned to build a database of different fracture models for teaching purposes in the long term. This database could grant access to universities or slack printing groups to provide models for local courses. As 3D printers, as well as the raw materials, are getting more and more affordable models like these can be produced very cost-efficiently [10].

In addition, in clinical practice, such 3D models can help understand of the ideal surgery and approaches by planning the screw osteosynthesis. The understanding of the fracture is therefore utmost importance for the decision of the treatment [12–14, 20]. While in some fractures, e.g. the twoplane fracture, an open reduction of the fracture from anterolateral and screw osteosynthesis are mostly the right choice [17, 20]. In triplane 1 fractures, there are already two directions for screw osteosynthesis: The more or less standardized anterior–posterior metaphyseal screw and the more oblique from the medial coming screw. Therefore, the exact fracture line is important to be recognized [17]. In triplane 2 fractures, the metaphyseal part is similar to the triplane 1 fracture. However, the 2 (!) fracture lines in the epiphysis might be difficult to judge, as shown in an earlier study of our group, which may lead to preoperative planning by 3D-printed models [21]. Thus, the screw positioning might be dependent on the epiphyseal fracture lines, which is a key issue to be understood [5, 12].

## Conclusion

The advantages of 3D-printed models in complicated fractures such as transitional fractures are obvious: The models can be easily obtained from already available and obtained sources (CT, MRI) and are technically easy to make with relatively low-cost effort to be used not only in education. 3d printing of fracture models will also arise as a promising tool in the future for operative planning as well as conservative therapy options.

## Supplementary Information

Below is the link to the electronic supplementary material.

Supplementary file1 (STL 1738 KB)

Supplementary file2 (STL 1417 KB)

Supplementary file3 (STL 6350 KB)

Supplementary file4 (STL 2027 KB)

Supplementary file5 (STL 1426 KB)

Supplementary file6 (STL 4351 KB)

Supplementary file1 (STL 1434 KB)
